# Metabolic and Dynamic Profiling for Risk Assessment of Fluopyram, a Typical Phenylamide Fungicide Widely Applied in Vegetable Ecosystem

**DOI:** 10.1038/srep33898

**Published:** 2016-09-22

**Authors:** Peng Wei, Yanan Liu, Wenzhuo Li, Yuan Qian, Yanxia Nie, Dongyeop Kim, Mengcen Wang

**Affiliations:** 1 Institute of Pesticide and Environmental Toxicology, Zhejiang University, No. 866 Yuhangtang Road, Hangzhou 310058, China; 2 South China Botanical Garden, Chinese Academy of Sciences, No. 723 Xingke Road, Guangzhou 510650, China; 3 Biofilm Research Labs, Divisions of Pediatric Dentistry & Community Oral Health, University of Pennsylvania, PA 19104, USA

## Abstract

Fluopyram, a typical phenylamide fungicide, was widely applied to protect fruit vegetables from fungal pathogens-responsible yield loss. Highly linked to the ecological and dietary risks, its residual and metabolic profiles in the fruit vegetable ecosystem still remained obscure. Here, an approach using modified QuEChERS (Quick, Easy, Cheap, Effective, Rugged and Safe) extraction combined with GC-MS/MS analysis was developed to investigate fluopyram fate in the typical fruit vegetables including tomato, cucumber, pepper under the greenhouse environment. Fluopyram dissipated in accordance with the first-order rate dynamics equation with the maximum half-life of 5.7 d. Cleveage of fluopyram into 2-trifluoromethyl benzamide and subsequent formation of 3-chloro-5-(trifluoromethyl) pyridine-2-acetic acid and 3-chloro-5-(trifluoromethyl) picolinic acid was elucidated to be its ubiquitous metabolic pathway. Moreover, the incurrence of fluopyram at the pre-harvest interval (PHI) of 7–21 d was between 0.0108 and 0.1603 mg/kg, and the Hazard Quotients (HQs) were calculated to be less than 1, indicating temporary safety on consumption of the fruit vegetables incurred with fluopyram, irrespective of the uncertain toxicity of the metabolites. Taken together, our findings reveal the residual essential of fluopyram in the typical agricultural ecosystem, and would advance the further insight into ecological risk posed by this fungicide associated with its metabolites.

In China, fruit vegetable as an important source of vitamins, minerals and fiber has taken up a large proportion in Chinese dietary consumption with a constant increasing tendency since the last decades^1^. However, fruit vegetables are generally cultivated in the greenhouse and the well-controlled environments with relatively high temperature and humidity always lead to severe suffering from fungal pathogens such as Deuteromycotina and Ascomycotina. Various chemical fungicides have been thus applied to reduce the yield loss caused by fungal diseases, particularly those with broad-spectrum antifungal activities^2^. Fluopyram, a typical pyridinyl moiety-contained phenylamide fungicide (Fig. 1) with the broad-spectrum activity, was developed by Bayer Crop Science in 2010^3^,^4^, and has been widely applied to eliminate fungal pathogens from fruit vegetables since its full registration in 2012 in China^5^. Broad-spectrum activity of this fungicide was owing to its inhibition on the ubiquitous enzyme succinate dehydrogenase (SDH) that functions as a ubiquitous and key element for mitochondrial electron transportation in the tricarboxylic acid cycle of fungal pathogens^6^,^7^.

In spite of the compensation of yield loss, food safety issues and ecological risks conversely caused by the excessive application of fungicides in vegetables have been raised as public concerns recently worldwide^2^,^8^. Fluopyram was classified as a low toxic compound due to the relatively low LD_50_ towards mammals (for rats, >2000 mg/kg bw), but it was also known to act as an endocrine disrupting compound (EDC) to exert side effects on the endocrine systems of human beings and wildlife at trace level^9^,^10^,^11^. For instance, it could induce tumor formation in the liver and thyroid of the rat by activation of CAR/PXR nuclear receptor^10^,^12^. More recently, it was also found that this fungicide was capable of altering the microbial diversity in soil ecosystem, leading to depraved soil health^13^. It seems that constant and excessive application of fluopyram may pose a risk towards human health through the imbalance of the associated agricultural ecosystem.

As a matter of fact, ecological and dietary risks caused by agrochemical residues were deemed to be highly linked to its side effects on the living creatures during the passed decades, and spectrum approaches-based disclosure of the residual profiles of agrochemical thus became an essential component and powerful tool in risk assessment. Up to date, analysis of residual fluopyram in plant matrix has been performed via gas chromatography (GC) and GC-MS, but only limited to watermelon and cucumber^5^,^14^, in which different procedures adopted for extraction and purification were inconvenient, and their applicability for diverse matrices are also unknown^5^,^14^. Furthermore, either fluopyram or its possible metabolite in agricultural ecosystem, especially in the vegetable ecosystems was largely unreported. Hence, a reliable, unified and compatible methodology remained to be developed for rapid qualitative and quantitative analysis of fluopyram and the related metabolism in such vegetable ecosystems.

In this work, we conducted field trials under the environment-controlled greenhouses towards the typical fruit vegetables^15^ of China such as tomato, cucumber and pepper, and meanwhile QuEChERS (Quick, Easy, Cheap, Effective, Rugged and Safe) extraction^16^ combined with gas chromatography-tandem mass spectrometry (GC-MS/MS) approach was developed to elucidate the residual profiles of fluopyram in these vegetable greenhouse ecosystems for assessment of the dietary risk. Furthermore, three metabolites previously unreported were identified during the rapid decline of fluopyram in the edible parts of vegetables and greenhouse soil. Our data provide a highly-compatible tool for monitoring trace amounts of fluopyram in plant- and environmental origin, and disclosure of the fate of fluopyram in the fruit vegetables ecosystems would significantly promote the further understanding of ecological and dietary risks posed by this fungicide and its metabolites.

## Results and Discussion

### Instrument optimization for analysis of fluopyram

**Figure 2:**
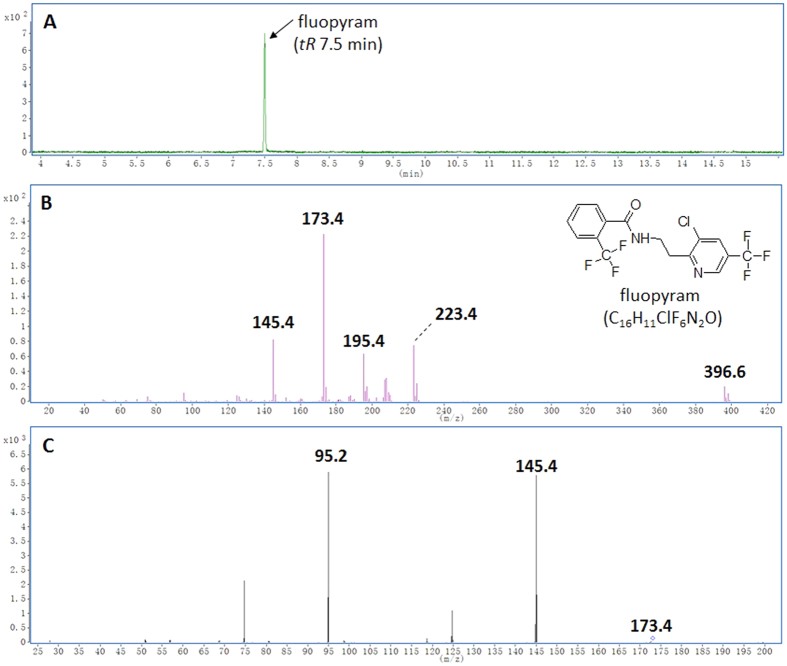
Total ion chromatogram (TIC) (**A**) and the GC-MS/MS elucidation of authentic fluopyram (**B**). Fluopyram (1 μg/kg) was detected at 7.5 min, and quantitative and qualitative ion pairs were acquired under MRM mode (**C**).

During the optimization of the GC-MS/MS method, authentic fluopyram detected at *tR* 7.5 min with the parent ion m/z 396.6 was monitored in the full scan mode in the range m/z 50–500 (Fig. 2A,B), and ion 173.4 was selected as the first-order parent ion fragments (Fig. 2B). Daughter scan mode was used for selection of the optimal quantitative and confirmative ions through continuous commissioning collision energy. The mass spectrometry conditions were carefully selected to provide a compromise solution between sensitivity, selectivity and structural information for quantitation purposes. Typically, the most intense ion in the MS or MS/MS spectrum was chosen as the parent ion or daughter ion, but if this ion had a low mass it might be better to select a less intense ion at a higher mass. Ultimately, we established MRM condition: MRM transitions were 173.4 > 145.4 (quantitation, collision energy, CE, 30 V) (Fig. 2C) and 173.4 > 95.2 (quantification, CE, 30 V) (Fig. 2C).

### Quality assurance and quality control

The matrix-matched calibration was done by the standard addition method in matrix extracts and the calibration curve of fluopyram was constructed by plotting analyte concentration again peak area. In the range of concentrations (from 1 to 5000 μg/kg), good linearity was achieved for the target compound with correlation coefficients higher than 0.99 for all standard solutions (Table 1).

The LOQs, defined as a S/N of 10:1, were 0.1 μg/kg in all matrixes tested in this work, while in the methods previously reported the LOQs for analysis of fluopyram in cucumber and watermelon were 3 μg/kg and 10.2 μg/kg^5^,^14^, respectively, which indicates the higher compatibility and sensitivity of the method currently developed.

### Analytical approach performance

**Figure 3:**
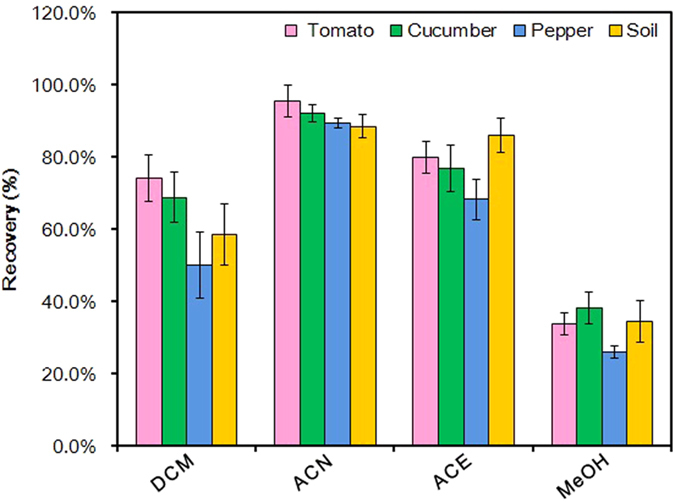
Comparison of recoveries obtained with different sample extraction solvent. Values are means ± standard deviations (shown by error bars) (*n* = 5).

For non-polar and polar pesticides, classic methods such as Mills method^17^ and Luke method^18^ were generally adopted. To determine the favorable extraction solvent, acetone (ACE), acetonitrile (ACN), dichloromethane (DCM) and methanol (MeOH) were accordingly tested for comparison of the recoveries. As a result, the recoveries of fluopyram in three different vegetables and soil samples obtained with ACE, DCM and MeOH were lower than ACN (Fig. 3), indicating that ACN was the ideal extraction solvent. As previously reported, ACN was a key component in QuEChERS that was first developed by Anastassiades *et al*. in 2003^19^ and widely applied for extraction and purification of a variety of chemicals from various matrices such as fruit, vegetable, soil, and meat^20^,^21^. Considering another advantage of QuEChERS that salting-out effect could raise recoveries of polar compounds^22^, we adopted QuEChERS-based approach for extraction and purification.

According to the modified QuEChERS, spiked recoveries at three spiking levels for tomato, cucumber, pepper and soil sample matrices were obtained as 90.5–95.6%, 92.0–93.2%, 89.0–95.4%, and 88.5–95.1%, with corresponding RSD values of 3.7–8.6%, 2.5–3.9%, 2.5–4.5% and 2.3–9.5%, respectively (Table 2). Besides, the intra-day and inter-day variability were lower than 10.7 and 13.5%, respectively at all spiked level. These data suggested the method established was of favorable performance and reliability (Table 3), and applicable for detection of trace amounts of residual fluopyram in fruit vegetable-associated samples.

### Initial deposition and dynamics of fluopyram

**Figure 4:**
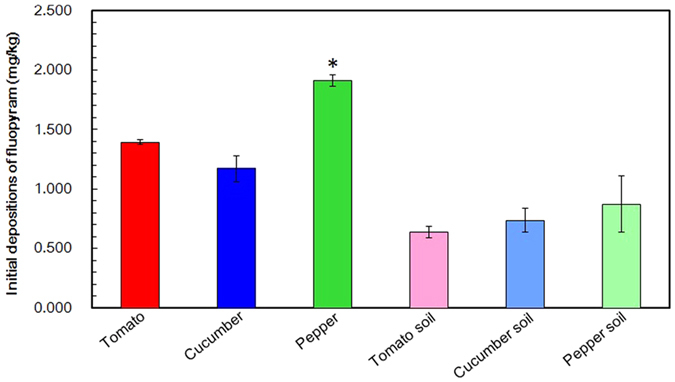
Initial deposits of fluopyram in the three fruit vegetables and soils. Values are means ± standard deviations (shown by error bars) (*n* = 3). *P < 0.05 as determined by Student’s *t-*test.

The edible parts of the three vegetables were collected after the foliar application. GC-MS/MS-based analysis showed that initial deposits of fluopyram followed the decreasing order of pepper > tomato > cucumber (Fig. 4). The initial deposit of fluopyram was 1.913 mg/kg in pepper, while the initial deposits in tomato and cucumber were 1.391 mg/kg and 1.172 mg/kg, respectively (Fig. 4). We found that individual pepper has the comparable size (superficial area) with individual tomato and cucumber, indicating the similar receipt of fraction of fluopyram applied, but the former was of the relatively lower water content in contrast to the latters (data unpublished), leading to the relatively lower fresh weight of individual pepper, namely lower biomass. Since the initial deposition was inversely proportional to the biomass of the plant^23^,^24^, it was proposed that the higher deposits of fluopyram in pepper were largely attributed to its lower biomass.

Compared with the edible part of vegetables, it was not surprising that almost 2-fold lower initial deposits of fluopyram were observed in the related greenhouse soil, which were 0.637, 0.738 and 0.872 mg/kg in tomato soil, cucumber soil and pepper soil (Fig. 4). It was attributed to that majority of the fluopyram were deposited on the surface of the fruit and leaf rather than the soil owing to the foliar application and high leaf area index^24^.

**Figure 5:**
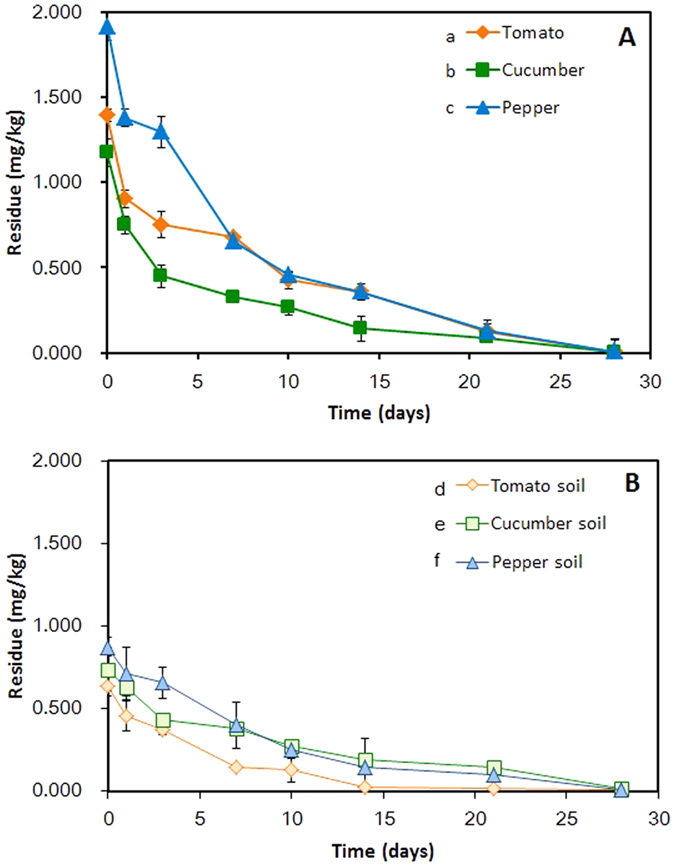
Dissipation curve of fluopyram. Fluopyram dissipated in fruit vegetables (**A**) including tomato (a), cucumber (b), pepper (c) and relevant soils (**B**) of tomato (d), cucumber (e), pepper (f). Values are means ± standard deviations (shown by error bars) (*n* = 3).

The dynamics curves demonstrated that the residual fluopyram dissipated rapidly within 7 days and persisted in three vegetables for an extended period of time (Fig. 5 and Table 4). Half-lives of fluopyram were calculated as 3.8 d in pepper, 3.9 d in cucumber and 4.4 d in tomato, indicating that the similar half-lives of fluopyram in the three vegetables, despite of the relatively higher degradative rate in pepper than in cucumber and tomato (Table 4). In addition, fluopyram declined constantly with half lives of 4.2, 5.7 and 4.3 d in greenhouse soil of tomato, cucumber and pepper, respectively (Table 4), showing the similar tendency as observed in the edible part of the plants (Fig. 5). Considering that the half life of fluopyram was from 3.8 d to 5.7 d, it was deemed to belong to the readily degradable fungicide in the vegetable ecosystem^2^,^25^.

Previously, the half-lives of fluopyram in watermelon and watermelon soil under a open ground were reported as 6.5–6.6 d and 15.8–24.8 d, respectively^5^, which were much higher than 3.8–5.7 d observed in our trial (Table 4). This seemed to be largely attributed to relatively high temperature and humidity of the greenhouse ecosystem, leading to the acceleration of the decline process^26^,^27^. Besides, in contrast to the open ground, the favorable condition of greenhouse provides a more profitable hotbed not only for the indigenous rhizomicroflora but also for epiphytes and endophytes^28^,^29^,^30^, which were considered to dominant biological factors contributing to synergic effect on agrochemical degradation^28^,^29^,^31^,^32^.

### Metabolic pathway of fluopyram

**Figure 6:**
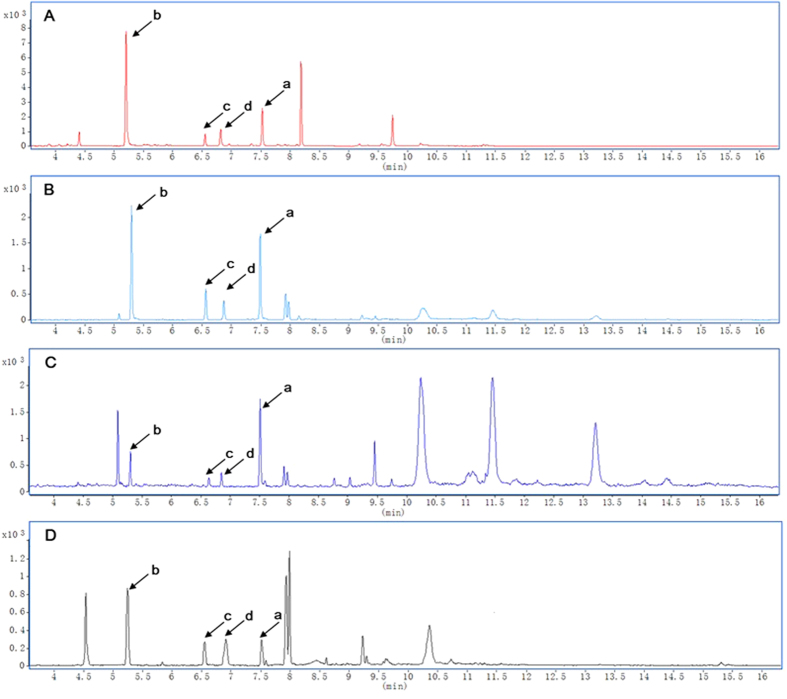
TIC of different samples obtained from GC-MS/MS using full scan mode for screening of metabolites of fluopyram. Fluopyram (a) was detected with retention at 7.5 min, and three peaks highly associated with fluopyram were detected at *tR* 5.3 min (b), 6.6 min (c) and 6.8 min (d) in tomato (**A**), cucumber (**B**), pepper (**C**) and greenhouse soil (**D**).

**Figure 7:**
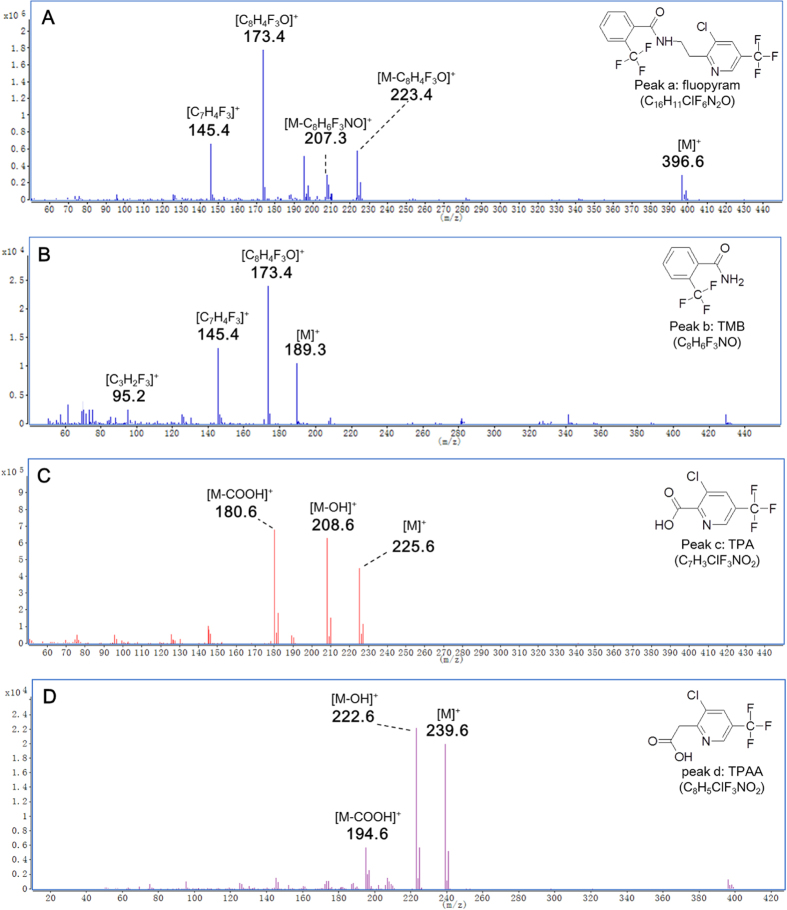
The characteristic fragmentations of fluopyram and the three metabolites. In association with fluopyram (**A**), structure elucidation lead to identification of the three metabolites as TMB (**B**), TPA (**C**) and TPAA (**D**).

During the rapid dissipation of fluopyram in the three vegetable greenhouses, a wide array of peaks in various samples was also observed through the full scan mode of GC-MS/MS (Fig. 6). Several peaks increased regularly along with the decline of fluopyram, but not observed in control group, were seemed to be highly associated with fluopyram. To clarify this issue, these relevant peaks were further selected and subjected to GC-MS/MS analysis per characteristic fragmentation and assigned by Agilent Mass-hunter Library NIST11.L database. Consequently, 3 peaks (Fig. 6) identified as fluopyram metabolites as follows (Fig. 7).

The mass fragments of 173.4 and 145.4 m/z existed in both of peak a and b, indicating that peak a and b had identical functional groups (Fig. 7A,B), suggesting peak b was originated from peak a. Instead of the absence of mass 396.6 and 207.3 m/z in fluopyram, mass of 189.3 emerged in peak b (Fig. 7B), which showed the loss of a moiety of 207.3 m/z from m/z 396.6. It was thus elucidated that a cleavage of fluopyram into a trifluoromethyl benzamide molecule (peak b) and a trifluoromethyl pyrimidine-containing molecule. Peak b was further identified as 2-trifluoromethyl benzamide (TMB) via the characteristic fragmentation mentioned above and database deconvolution (Fig. 7B and Table S1). Interestingly, the trifluoromethyl pyrimidine-containing molecule speculated as 2-(3-chloro-5-(trifluoromethyl)pyridin-2-yl)ethanol (TPE) was not observed in all samples collected, while the peak c and d identified as 3-chloro-5-(trifluoromethyl)picolinic acid (TPA) (Fig. 7C and Table S1) and 3-chloro-5-(trifluoromethyl)pyridine-2-acetic acid (TPAA) (Fig. 7D and Table S1) seemed to be the degraded products of TPE.

**Figure 8:**
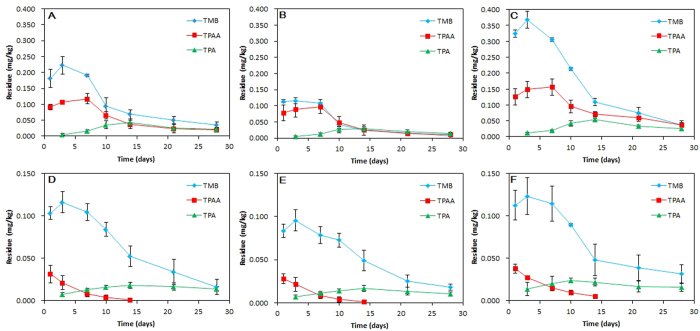
Distribution and formation dynamics of fluopyram metabolites in the fruit vegetable ecosystem. TMB, TPA and TPAA were simultaneously analyzed in tomato (**A**), cucumber (**B**), pepper (**C**), tomato soil (**D**), cucumber soil (**E**) and pepper soil (**F**). Values are means ± standard deviations (shown by error bars) (*n* = 3).

For the further elucidation of the metabolic pathway of fluopyram, we also quantitatively analysed distribution and formation of these metabolites in vegetables and soils. As a result, we found TMB began to occur at 1 d and then gradually dissipated from 3 d to 28 d in both vegetables and soils, and finally maintained at the level from 0.008 mg/kg to 0.036 mg/kg (Fig. 8). Similar to TMB, the formation dynamics of TPAA showed an increasing tendency from 1d to 7 d in three vegetables and followed by a constant decline until 28 d (Fig. 8). However, in the soils, TPAA exhibited a constant decline curve and became undetectable (<0.001 mg/kg) at 14 d (Fig. 8). Highly associated with TPAA, TPA began to occur when TPAA significantly declined within 14 d, and followed by a slight decline stage and finally maintained at a relatively stable level until 28 d (Fig. 8).

**Figure 9:**
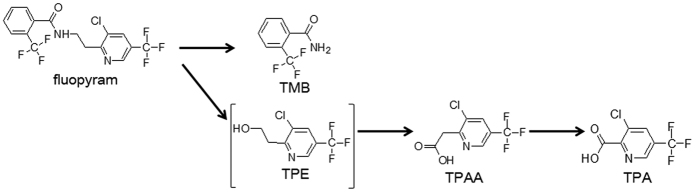
The proposed metabolic pathway of fluopyram in the fruit vegetable ecosystem. Square brackets indicate that TPE is an intermediate speculated.

Based on the qualitative and quantitative analyses mentioned above, it was deduced that the parent molecule was split into TMB and the tentative TPE in the initial step of fluopyram metabolic pathway, despite that the bond between imino and methylene was not chemically active. Interestingly, we found that fluopyram was degraded in the sterilized soil with a 40-fold higher half-lives than the field trial, and neither TMB nor TPE was detected (data not shown). It was likely that microbial enzymatic catalysis in the vegetable ecosystem was responsible for this cleveage reaction at the single carbon-nitrogen bond of fluopyram^33^,^34^. Additionally, the missing link converting fluopyram to TPAA mediated by TPE could be explained by the rapid oxidative transformation of TPE to TPAA (Fig. 9), which was further decarboxylated and oxidized to generate TPA (Fig. 9)^35^. Similar to the microbial cleavage of nitrogen-carbon bond, indigenous microbial transformation was possibly a predominant factor directing the decarboxylation and oxidization in the downstream of the metabolic pathway (Fig. 9).

Taken together, cleavage of the parent molecule fluopyram to produce the TMB, TPA and TPAA was the ubiquitous metabolic pathway of fluopyram in the three vegetable greenhouses, among which TMB was the primary metabolite whereas TPA and TPAA were secondary metabolites (Fig. 9).

### Incurrence and dietary risk of fluopyram

Although the rapid decline pattern of fluopyram was observed under a single application in the dynamics trial, its incurrence level after multi-application remained to be elucidated for assessment of the dietary risk. At pre-harvest interval (PHI) 7 days, the incurrence of fluopyram in three vegetables were followed the decreasing order of pepper (0.1603 mg/kg) > tomato (0.0769 mg/kg) > cucumber (0.0571 mg/kg) (Table 5). At PHI 14 d and 21 d, the incurrence of fluopyram in cucumber decreased to 0.0043–0.0303 mg/kg. In pepper and tomato, the incurrence of fluopyram at PHI 14 d and 21 d ranged from 0.0108–0.1603 mg/kg and 0.0058–0.0331 mg/kg, respectively (Table 5). Incurrence level were decreased along with the duration of PHI and highest residue (HR) of fluopyram in the three vegetables were all found at PHI 7 d. According to the calculation of hazard quotient (HQ) based on HR value (Table 5) and GEMS/Food consumption database (fruit vegetable consumption in China)^36^, HQs in three fruit vegetables were all lower than 1 (Table 5), indicating safety of the fruit vegetable incurred with residues of this fungicide to the consumers by daily consumption. However, we found that the edible parts of three vegetables were incurred with TMB, TPA and TPAA at the trace level (Tables S2, S3 and S4). It is still remained obscure whether the three metabolites of fluopyram should be considered in the dietary risk assessment since the unknown toxicological effect of these metabolites. Therefore, the further elucidation of the toxicological effect on human beings needs to be focused through the short-term and long-term toxicological tests.

## Conclusion

An effective and sensitive analytical method using a modified QuEChERS extraction with GC-MS/MS analyses was developed to detect the trace amounts of fluopyram in the three fruit vegetables (tomato, cucumber, pepper) and relevant soils. After the single application at 62.4 g a.i. ha^−1^, fluopyram dissipated rapidly in the vegetable greenhouse ecosystem in accordance with the first-order rate dynamics with the maximum half-life as 5.7 d, in which the relatively high initial deposition and degradative rate were both observed in the pepper. As a readily degradable fungicide, fluopyram was split into a primary metabolite TMB and followed by formation of two secondary metabolites TPAA and TPA along with the decline process, which occurred as its ubiquitous metabolic pathway in the vegetable greenhouse ecosystem. After the multi-application, the incurrence of fluopyram in tomato, cucumber and pepper at PHI 7–21 d was from 0.0108 to 0.1603 mg/kg, and all the related HQs were below 1, indicating the temporary safety on consumption of the fruit vegetables incurred with fluopyram, irrespective of the uncertain toxicological effects of the metabolites. Taken together, we provide a highly-compatible tool to monitor fluopyram in plant- and environmental origin, and the current elucidation of residual essential of fluopyram in the typical agricultural ecosystem would advance the further insight into risks posed by this fungicide associated with its metabolites.

## Materials and Methods

### Chemicals and reagents

Authentic fluopyram (purity 99.4%) was purchased from Dr. Ehrenstorfer (Augsburg, Germany) and fluopyram SC (500 g/L) used for field trial was obtained from Institute for the Control of Agrochemicals, Ministry of Agriculture, China. Ultrapure water was purified using Pall Cascada AN (Pall Corporation, USA). The one-off membrane (0.22 μm, polytetrafluoroethylene, PTFE) microfilter (MITEX, Millipore, USA) were applied for filtration of the analyte. ACN (Acetonitrile, HPLC grade), EtOAc (ethyl acetate, GC grade) and other organic solvent were purchased from Sigma-Aldrich (USA). Magnesium sulfate, sodium acetate and PSA (primary secondary amine) were purchased from Waters (USA).

### Field trial

The three selected fruit vegetables were tomato (*Lycopersicon esculentum* cv. Dahongyihao), cucumber (*Cucumis sativus L.* cv. Shandongmici) and pepper (*Capsicum annuum L.* cv. Qingjiaowang), for which field trial were conducted in the greenhouses locating at the Experiment Base (31.321°N, 119.552°E, Jiangdu District, Jiangsu Province) of Institute of Pesticide and Environmental Toxicology, Zhejiang University, from July to September, 2015. All processes and operations in the supervised trials were strictly carried out per Good Agricultural Practices (GAPs) issued by the Institute for the Control of Agrochemicals, Ministry of Agriculture, China, and any endangered wildlife specie or protected area of land or sea was not involved in this trial.

For each vegetable, each treatment consisted of three adjacent replicate plots with complete randomized block design (CRD), each with an area of 15 m^2^ and the control were sprayed with water only. The greenhouse soil was consisted of 50% of sand, 17% clay, 31% silt and 2% organic matter with pH at 6.1, and classified to sandy loam^37^. To investigate the initial deposition, dynamics and metabolism of fluopyram, fluopyram SC was sprayed at 62.4 g a.i. ha^−1^ (double of the maximum recommended dosage) when the first fruit on the main stem reached the typical size and form. Vegetables (edible part) and soils (at least 500 g) were taken at 2 h, 1, 3, 7, 14, 21 and 28 day using five point sampling method. For monitoring of the incurrence, fluopyram was sprayed at 31.2 g a.i. ha^−1^ (the maximum recommended dosage) for three times with an interval of 7 days. Vegetables (edible part) were collected during the maturity period at pre-harvest interval (PHI) 7, 14 and 21 days after the last application and stored in a freezer after quartering and homogenized into the sample vial. Tiny stones and other unwanted materials were removed from soil samples and then screened through 40-mesh sieves. All the samples pretreated were stored in a freezer at −20 °C until analysis.

### Extraction and purification of fluopyram

The modified QuEChERS was done as follows. Twenty gram of homogenized vegetable sample (or 10 g of soil sample) was weighed and mixed in a 250 mL-centrifuge bottle with 50 mL ACN and 10 mL deionized water. The mixture was shaken on an automatic horizontal shaker at 180 rpm for 30 min at room temperature to fully disperse the sample. After centrifugation at 4000 rpm for 5 min, the aliquot of the extract (25 mL) was transferred into a 50-mL centrifuge tube. After the addition of 6 g magnesium sulfate and 1.5 g sodium acetate, each mixture was shaken vigorously for 1 min and centrifuged at 4000 rpm for 5 min. The supernatant (25 mL) was concentrated by a rotatory evaporator at 45 °C for further purification. The resulting concentrates were re-dissolved with ethyl acetate (2 mL) and pipetted into a 2 mL-clean up tube filled with 50 mg PSA and 150 mg magnesium sulfate. After shaking vigorously for 30 s, the tube was centrifuged at 12,000 rpm for 5 min. After filtration through a 0.22 μm filter, the resulting filtrates were subjected to quantitative analysis of fluopyram.

### Qualification and quantification of fluopyram

Authentic fluopyram was dissolved in EtOAc at 500 mg/kg as the stock solution and stored at −10 °C. A series of dilutions of fluopyram in EtOAc spiked at the concentrations 0.001, 0.01, 0.1, 1 and 5 mg/kg in the matrices were used as the working solutions for quantification of the fluopyram using external standard method. All of the solutions were stored at 4 °C in the dark.

For quantification of fluopyram, an Agilent HP-5 MS (30 m × 0.25 mm i.d., with 0.25 μm film thickness) capillary column was installed in a GC-MS/MS (Agilent 7000 C). Helium (99.999%) was applied as the carrier gas at a constant flow of 2.25 mL/min and Nitrogen (99.999%) at rate of 1.5 mL/min was used as collision gas. The temperature of injector was set at 280 °C, and the injection volume was 1 μL in the splitless mode. The oven temperature was raised from 80 °C at a rate of 30 °C min^−1^ to 220 °C and held for 1 min, and then raised to 240 °C at a rate of 5 °C min^−1^ and held for 0 min, and then raised to 260 °C at a rate of 10 °C min^−1^ and held for 4 min. MS/MS was operated in electron ionization mode using electron impact ionization (EI, 70 eV) and detector voltage of 1.1 kV with a mass range of 50~500 m/z. Multiple reaction monitoring (MRM) transitions were 173.0 > 145.4 (quantitation, collision energy, CE, 30 V) and 173.0 > 95.2 (identification, CE, 30 V). Separation was performed on an Agilent HP-5 MS (30 m × 0.25 mm i.d., with 0.25 μm film thickness) capillary column. The temperature of transfer line and ionization source was maintained at 250 °C and 230 °C, respectively. The retention time (*tR*) was 7.5 min. The software Agilent 7000 Mass Hunter was applied for instrument control, data acquisition and processing.

### Methodological performance and reliability

The fluopyram-free vegetables (tomato, cucumber, pepper) and greenhouse soil samples were fortified with fluopyram at 0.001, 0.05 and 0.5 mg/kg with 5 replicates. Spiked samples were left to stand for 60 min to allow pesticide absorption onto the sample adequately, and then subjected to treatment and analysis under the same conditions as described above. Recovery was calculated for evaluation of the method performance.

To evaluate reliability of the method developed, RSD_R_ (intra-day precision) was measured for the repeatability by comparing standard deviation of the recoveries of five replicates in the same day, and RSD_R_ (inter-day precision) was determined by analyzing spiked samples in three alternate days for the reproducibility^38^. Intra- and inter-day precisions tests were also done at 0.001, 0.05 and 0.5 mg/kg with nine replicates on 3 different days.

### Dynamics fitting

The dynamics of fluopyram in various samples was analysed by plotting the residue concentration against time via the first-order rate equation:
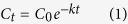
*C*_t_ and *C*_0_ represent the concentrations of the residual fluopyram at the day *t* and day 0 (2 h), respectively, and k is the dissipation rate constant. The half-life (t_1/2_) is defined as the time required for the pesticide residue level to fall to the half of the initial residual level of day 0 (*i.e.*, *C*_0_) and calculation was done using the following equation^2^:
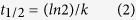


### Track of fluopyram metabolism

For screening of the potential metabolites of fluopyram, the samples collected from dynamics trial were subjected to GC-MS/MS using the full scan mode. The peaks in the total ion chromatogram (TIC) highly representing increased tendency along with the dynamics of fluopyram were further isolated for characterization of the molecular structure. The parent ions of the tentative metabolites were selected and further subjected to MS/MS using daughter scan mode for characteristic daughter ion fragmentation. Finally, identification of the metabolites’ structure was done by parent ion deconvolution and the daughter ion fragmentation assignment through the Agilent Mass-hunter Library NIST11.L database. To determine the concentration of the proposed metabolites in the vegetables and soils, extraction and purification procedure were performed per the analytical process for fluopyram. For each metabolite, a pair of quantitative ions were selected for quantification with the use of *p*-tert-butylphenol as an internal standard (Table S1).

### Dietary risk assessment

For assessment of the dietary risk of fluopyram, the estimated daily intake (EDI) are expressed as a percentage of the the acceptable daily intake (ADI) for a 60-kg adult person, and the ADI for fluopyram is 0.01 mg/kg body weight (bw)/day^39^. EDI was calculated by multiplying the highest residue (HR) in each sample (mg/kg) with the average daily per capita consumption estimated for fruit vegetables of 58.291 g/day in China^36^. The risk assessment of intakes compared to pesticide toxicological data was conducted via calculation of the hazard quotient (HQ), where EDI was divided by the relevant ADI^40^. The relevant equations were as follows:
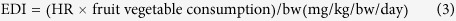

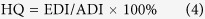


It would indicate an unacceptable risk if the HQ calculated is higher than 1, and the higher HQ value represents the higher risk, whereas the dietary risk is acceptable if the HQ calculated is lower than 1^41^.

## Additional Information

**How to cite this article**: Wei, P. *et al*. Metabolic and Dynamic Profiling for Risk Assessment of Fluopyram, a Typical Phenylamide Fungicide Widely Applied in Vegetable Ecosystem. *Sci. Rep.* **6**, 33898; doi: 10.1038/srep33898 (2016).

## Supplementary Material

Supplementary Information

## Figures and Tables

**Figure 1:**
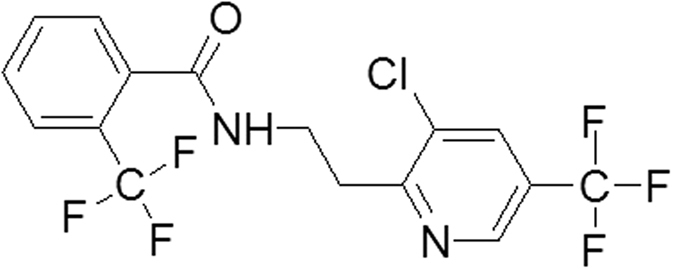
The chemical structure of fluopyram.

**Table 1:** Relative standard deviation of retention time for fluopyram.

Matrix	Rt^a^ RSD(%) (*n* = 10)	Calibration equation	Slope RSD(%) (*n* = 3)	R^2^
Tomato	0.84	*Y* = 16222*C* + 430.18	4.1	0.9993
Cucumber	0.78	*Y* = 18223*C* + 425.15	4.3	0.9995
Pepper	0.99	*Y* = 17026*C* + 443.21	7.2	0.9992
Soil	1.10	*Y* = 17922*C* – 1080.3	6.9	0.9972

Calibration equation in different matrices at 1–5000 μg/kg concentration range.

^a^Retention time.

**Table 2:** The recovery experiments with various matrices and levels for evaluation of method performance.

Spiking level (mg/kg)	Tomato		Cucumber		Pepper		Soil	
	Recovery (%)	RSD (%)	Recovery (%)	RSD (%)	Recovery (%)	RSD (%)	Recovery (%)	RSD (%)
0.001/0.005*	90.5 ± 7.8	8.6	92.0 ± 2.7	3.0	89.0 ± 2.2	2.5	90.7 ± 8.6	9.5
0.01/0.05	95.6 ± 3.5	3.7	92.2 ± 2.3	2.5	89.5 ± 2.4	2.6	88.5 ± 4.0	4.5
0.5/1	91.9 ± 4.3	4.7	93.2 ± 3.6	3.9	95.4 ± 4.3	4.5	95.1 ± 2.2	2.3

*0.001/0.005 expresses the fortified level of three vegetables/the fortified level of soil (*n* = 5).

**Table 3:** The recovery experiments with intra-day and inter-day variability for evaluation of method reliability.

RSD_R_ (%)	Spiking 0.001/0.005* mg/kg		Spiking 0.01/0.05 mg/kg		Spiking 0.5 /1 mg/kg	
	Intra-day^a^	Inter-day^b^	Intra-day^a^	Inter-day^b^	Intra-day^a^	Inter-day^b^
Tomato	5.9	9.7	6.1	10.2	4.	8.5
Cucumber	4.6	8.5	6.7	13.5	7.0	7.9
Pepper	6.2	11.9	4.8	9.2	7.2	6.3
Soil	5.3	10.6	3.6	8.8	10.7	5.1

*0.001/0.005 expresses the fortified level of three vegetables/the fortified level of soil (^a^ *n* = 3, ^b^ *n* = 9).

**Table 4:** Dynamics equations, correlation coefficients and half-lives of fluopyram in three fruit vegetables and soils.

Sample	Dynamic equation	Correlation coefficient (R^2^)	Half-live(d)
Tomato	*C*_t_ = 1.632e^−0.158t^	0.8799	4.4
Cucumber	*C*_t_ = 1.165e^−0.177t^	0.8820	3.9
Pepper	*C*_t_ = 2.368e^−0.181t^	0.9056	3.8
Tomato soil	*C*_t_ = 3.696e^−0.166t^	0.9619	4.2
Cucumber soil	*C*_t_ = 0.814e^−0.121t^	0.8789	5.7
Pepper soil	*C*_t_ = 1.108e^−0.163t^	0.8964	4.3

**Table 5:** Incurrence, HR and HQ of fluopyram in edible parts of the three fruit vegetables.

Sample	Crop group*	Incurrence (mg/kg)			HR (mg/kg)	HQ
		PHI 7 d	PHI 14 d	PHI 21 d		
Tomato	8–10B	0.0769	0.0331	0.0058	0.0769	0.4483
Cucumber	8–10C	0.0571	0.0303	0.0043	0.0571	0.3328
Pepper	8–10B	0.1603	0.0861	0.0108	0.1603	0.9344

*Code of Federal Regulations Title 40 Part 180.41 Crop group table (40 CFR 180.41). Group 8–10B and 8–10C indicate the fruit vegetable group.
