# Corrigendum: Metabolic and Dynamic Profiling for Risk Assessment of Fluopyram, a Typical Phenylamide Fungicide Widely Applied in Vegetable Ecosystem

**DOI:** 10.1038/srep41347

**Published:** 2017-01-25

**Authors:** Peng Wei, Yanan Liu, Wenzhuo Li, Yuan Qian, Yanxia Nie, Dongyeop Kim, Mengcen Wang

Scientific Reports 6: Article number: 3389810.1038/srep33898; published online: 09 22 2016; updated: 01 25 2017

This Article contains errors in the Materials and Methods section under subheading ‘Chemicals and reagents’.

“Authentic fluopyram (purity 99.4%) was purchased from Dr. Ehrenstorfer (Augsburg, Germany) and fluopyram SC (500 g/L) used for field trial was obtained from Institute for the Control of Agrochemicals, Ministry of Agriculture, China”.

should read:

“Fluopyram standard (99.4%, purity) was purchased from Dr. Ehrenstorfer (Augsburg, Germany) and fluopyram formulation applied in field trial was 500 g/L fluopyram • trifloxystrobin SC (suspension concentrate), a commercially available product manufactured by Bayer Crop Science”.

