# Supplementary material for: Metabolic and Dynamic Profiling for Risk Assessment of Fluopyram, a Typical Phenylamide Fungicide Widely Applied in Vegetable Ecosystem
Source: Sci Rep. 2016 Sep 22;6:33898. doi: 10.1038/srep33898 (PMC5031996; doi:10.1038/srep33898)
Supplement: Supplementary Information [file srep33898-s1.docx]

**Metabolic and Dynamic Profiling for Risk Assessment of Fluopyram, a Typical Phenylamide Fungicide Widely Applied in Vegetable Ecosystem**

Peng Wei^1^

Yanan Liu^1^

Wenzhuo Li^1^

Yuan Qian^1^

Yanxia Nie^2^

Dongyeop Kim^3^

Mengcen Wang^1^

^1^Institute of Pesticide and Environmental Toxicology, Zhejiang University, No. 866 Yuhangtang Road, Hangzhou 310058, China.

^2^South China Botanical Garden, Chinese Academy of Sciences, No. 723 Xingke Road, Guangzhou 510650, China.

^3^Biofilm Research Labs, Divisions of Pediatric Dentistry & Community Oral Health, University of Pennsylvania, PA 19104, USA.

Table S1. Key parameters for identification and quantification of the three metabolites including TMB , TPAA and TPA

| Name | Molecular formula | CAS number | Deconvolution  matching degree (%) | Quantitative  ion pair (m/z) | Mass-hunter library |
| --- | --- | --- | --- | --- | --- |
| TMB | C_8_H_6_F_3_NO | 360-64-5 | 98.92 | 189.3>145.3 | NIST11.L |
| TPA | C_7_H_3_ClF_3_NO_2_ | 80194-68-9 | 92.65 | 225.6>180.6 | NIST11.L |
| TPAA | C_8_H_5_ClF_3_NO_2_ | 1000522-34-8 | 93.39 | 239.6>194.6 | NIST11.L |
| PTBP* | C_10_H_14_O | 98-54-4 | 99.26 | 150.2>135.2 | NIST11.L |

**p*-tert-butylphenol selected as an internal standard (ISTD)

Table S2. Incurrence of TMB in edible parts of the three fruit vegetables

| Sample | Crop group* | Incurrence (mg/kg) | | | HR (mg/kg) |
| --- | --- | --- | --- | --- | --- |
|  |  | PHI 7 d | PHI 14 d | PHI 21 d |  |
| Tomato | 8-10B | 0.0563 | 0.0251 | 0.0094 | 0.0563 |
| Cucumber | 8-10C | 0.0358 | 0.0147 | 0.0066 | 0.0358 |
| Pepper | 8-10B | 0.0921 | 0.0432 | 0.0098 | 0.0921 |

*Code of Federal Regulations Title 40 Part 180.41 Crop group table (40 CFR 180.41). Group 8–10B and 8–10C indicate the fruit vegetable group.

Table S3. Incurrence of TPAA in edible parts of the three fruit vegetables

| Sample | Crop group* | Incurrence (mg/kg) | | | HR (mg/kg) |
| --- | --- | --- | --- | --- | --- |
|  |  | PHI 7 d | PHI 14 d | PHI 21 d |  |
| Tomato | 8-10B | 0.0311 | 0.0108 | 0.0024 | 0.0311 |
| Cucumber | 8-10C | 0.0224 | 0.0083 | 0.0019 | 0.0224 |
| Pepper | 8-10B | 0.0623 | 0.0284 | 0.0058 | 0.0623 |

*Code of Federal Regulations Title 40 Part 180.41 Crop group table (40 CFR 180.41). Group 8–10B and 8–10C indicate the fruit vegetable group.

Table S4. Incurrence of TPA in edible parts of the three fruit vegetables

| Sample | Crop group* | Incurrence (mg/kg) | | | HR (mg/kg) |
| --- | --- | --- | --- | --- | --- |
|  |  | PHI 7 d | PHI 14 d | PHI 21 d |  |
| Tomato | 8-10B | 0.0166 | 0.0052 | 0.0011 | 0.0166 |
| Cucumber | 8-10C | 0.0098 | 0.0034 | <0.0010 | 0.0098 |
| Pepper | 8-10B | 0.0294 | 0.0084 | 0.0023 | 0.0294 |

*Code of Federal Regulations Title 40 Part 180.41 Crop group table (40 CFR 180.41). Group 8–10B and 8–10C indicate the fruit vegetable group.
